# Environmental changes and risk of plague epidemics in Indonesia

**DOI:** 10.1371/journal.pntd.0013839

**Published:** 2025-12-30

**Authors:** Ristiyanto, Farida Dwi Handayani, Sitti Ganefa Pakki, Arief Mulyono, Muhammad Choirul Hidajat, Arum Sih Joharina, Fabien Marchois, Ni Luh Putu Indi Dharmayanti, Anung Sugihantono, Sylvie Manguin, Tofan Agung Eka Prasetya, Ira Nurmala, Sandra Perez, Triwibowo Ambar Garjito, Laurent Gavotte, Roger Frutos

**Affiliations:** 1 Research Center for Public Health and Nutrition, Health Research Organization, National Research and Innovation Agency Indonesia, Jakarta, Indonesia; 2 Eijkman Research Center for Molecular Biology, Health Research Organization, National Research and Innovation Agency Indonesia, Jakarta, Indonesia; 3 Zoonoses Working Group, Directorate Communicable Disease Control, Directorate General Disease Prevention and Control, Ministry of Health Indonesia, Jakarta, Indonesia; 4 National Laboratory for Environmental Health Surveillance, Salatiga, Indonesia; 5 University Côte d’Azur, Nice, France; 6 Health Polytechnic of Semarang, Ministry of Health Indonesia, Jakarta, Indonesia; 7 HydroSciences Montpellier (UMR-HSM), Institut de Recherche pour le Développement (IRD France), CNRS, Montpellier, France; 8 Faculty of Vocational Studies, Universitas Airlangga, Surabaya, Indonesia; 9 Faculty of Public Health, Universitas Airlangga, Surabaya, Indonesia; 10 UMR 228, Espace Dev, University of Montpellier, Montpellier, France; 11 Cirad, UMR 17, Intertryp, Montpellier, France; 12 Department of Pathology, Faculty of Medicine-Ramathibodi Hospital, Mahidol University, Bangkok, Thailand; 13 School of Public Health, Xiamen University, Xiamen, China; Institute of Continuing Medical Education of Ioannina, GREECE

## Abstract

**Background:**

Historical epidemiological data indicate that plague epidemics caused thousands of deaths in Indonesia between 1911 and 1956. During this period, silent phases of the disease were observed, followed by re-emergences several years or even decades later in certain regions. The Indonesian government, both at the regional and central levels (notably the Ministry of Health of the Republic of Indonesia), has undertaken decades of epidemic management efforts, including plague surveillance, medical treatments, vector control, and improvements in individual and environmental sanitation. There were a few sporadic outbreaks in 1968, 1987, and 2007. Since then, no further cases have occurred, but this could just be another silent phase.

**Methodology:**

A literature search comprising articles and reports including published and unpublished dissertations, was performed using the PubMed online database, the Directorate General of Disease Prevention and Control, the Ministry of the Health Republic of Indonesia, Institute for Vector and Reservoir Control Research and Development, The Ministry of Health of Indonesia, Provincial Health Offices, Google Scholar, Science Direct, Medicus Index for the Southeast Asian Region (IMSEAR) and co.IMSEAR), and others. All literature referring to plague in Indonesia (1923–2019) was used as a reference for this article. The Global Land Cover 1992–2020 from the European Space Agency database was used to monitor land cover changes with a spatial resolution of 300 meters. The Esri Sentinel-2 Land Cover Explorer database, created by ESRI (Environmental Systems Research Institute, Redland, California, USA) using Sentinel-2 satellite imagery was also used. This database shows global land cover change between 2017 and 2023, using only 2023 data. Seven land cover classes were identified: Water, Forest, Flooded fields, Fields, Urban, Bare ground, and Pasture.

**Findings:**

Environmental changes, essentially land conversion, have occurred in regions where plague outbreaks were previously recorded, with the exception of Eastern Java. Land conversion, increased human population density, and the heightened risk of human–rodent interactions could contribute to a resurgence of plague epidemics in Indonesia. Vectors and rodent hosts of *Yersina pestis* are still present in all regions but one with a higher human population density and thus a higher risk of contact. In Eastern Java, the environment remained the same as when plague outbreaks previously occurred.

**Conclusions:**

We conclude that the historical areas of plague outbreaks have a potential for silent periods of plague transmission which could last decades. Furthermore, land conversion and the development of human settlements in these regions have led to a higher human presence, thus potentially increasing the risk of contact and transmission. There is therefore today a risk of plague resurgence in Indonesia and the current plague-free period might just be a silent period. In order to prevent outbreaks of plague after a period of silence and to implement an early alert system of plague transmission from animals to humans via fleas, the monitoring of potential *Y. pestis* circulation in sylvatic areas also needs to be intensified.

## Author summary

Plague caused thousands of deaths in Indonesia from 1911 to 1986. Afterward, for more than 30 years there have been no bubonic or pneumonic plague reports in Indonesia. The Regional and Central Indonesian Government (Ministry of Health of the Republic of Indonesia) has undertaken decades of outbreak management such as plague surveillance, treatment, and vector control, as well as improvement of individual and environmental sanitation. As a consequence, the current incidence rate is zero. However, the assessment of results from the Ministry of Health indicates gaps in the implementation of surveillance in humans, animals, and laboratory capacity. Nevertheless, Indonesia is considered a country with a low risk of plague transmission. Climate change is predicted to localize the plague in certain areas, especially in the highlands. These areas, known as sylvatic areas, are inhabited by various populations of rats and fleas, which were confirmed to be either resistant or sensitive to *Yersinia pestis*. Some of which were confirmed to be either resistant or sensitive to *Y. pestis*. A silent period of plague transmission, which lasted decades, has previously been observed, thus raising questions. Environmental changes strongly impacted regions where plague outbreaks have been recorded in the past, with the exception of Eastern Java. However, vectors and rodent hosts involved in historical outbreaks are still present. Environmental changes have led to land conversion, a higher human population density, and a higher risk of human/rodent interactions. Although having impacted the natural environment, environmental changes may lead to a higher risk of future outbreaks of plague in Indonesia. The current lack of outbreaks might just be a silent period as already observed in the past.

## Introduction

Plague, one of the deadliest diseases in human history, is a bacterial zoonotic disease caused by *Yersinia pestis* and transmitted between animals or from animal to humans by infected rat fleas. There are more than 80 species of fleas worldwide that have been identified as vectors of plague, with *Xenopsylla cheopis* (the Oriental rat flea) being regarded as the principal vector [1]. This disease has caused three major human pandemics which led to the death of millions of people from the VIth to the XIXth Century [2]. Plague epidemics have occurred in Europe, Africa, North and South America, and Asia. Although the incidence of plague infection has only been reported on a small scale in some endemic countries, the potential for plague transmission is still present. The wide distribution of endemic animal hosts and vectors, resistant hosts passing the bacterium to their offspring, and widespread susceptible hosts are still a threat in many countries in Asia, Africa, and South America [3,4]. Urbanization, deforestation, mining, social conflict, and civil war have also contributed to the outbreaks in these regions [5].

**Fig 1:**
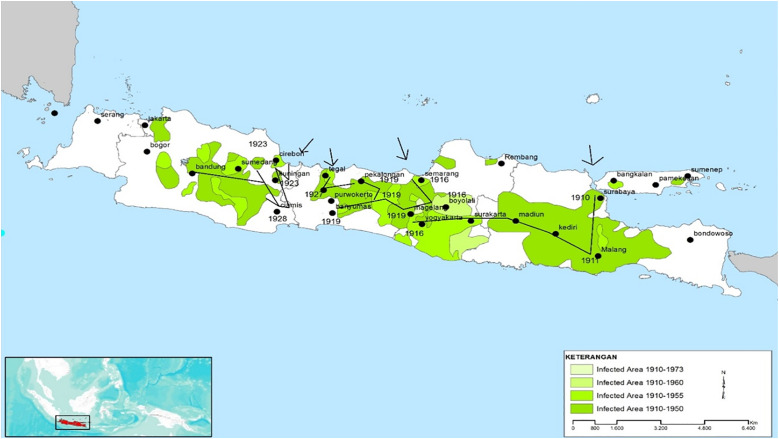
Distribution of plague in Java, Indonesia, 1910–1950. Map base layer: https://maps.arcgis.com/home/item.html?id=4ad930360ef64a828cf58a1a127c9a9d.

In Indonesia, plague was first documented in North Sumatra in 1905. Since then, the human plague has been reported to be endemic in several parts of Indonesia (Fig 1). From 1911 to 1939, large-scale bubonic plague epidemics were reported in East Java, Central Java, Yogyakarta, and West Java with a mortality rate reaching 70%–80% [6–8]. Despite experiencing several periods of silence, human cases of bubonic plague in Indonesia were still found until 2007. The persistence and recurrent plague outbreaks in Indonesia are likely caused by various factors resorting to biology, socio-economy, environment, and culture. Various actions have been undertaken to prevent and control the transmission of plague. Intensified and continuous surveillance of humans, rats, and fleas in endemic areas has been continuously carried out to ensure that there were no cases and deaths. Efforts have also been done to prevent the spread of plague from foci areas to non-endemic areas, to monitor previous outbreak areas and to prevent the entry of the disease from abroad [9].

Currently, transmission and new cases of plague are not reported in Indonesia. During the last decade, *Y. pestis* was no longer found in humans, rats, and fleas. However, routine plague surveillance is still ongoing. Over the last few decades, Indonesia has seen a lot of environmental changes in particular with respect to land use, land conversion, and increase of human settlements. Here, we review human plague cases and risk factors associated with its transmission in Indonesia from the early 1900s to the present to explain and conclude whether plague in Indonesia has been eradicated or is still in a silent period and could, at any time, have the potential for a resurgence.

## Source of data

A literature search was performed using the PubMed online database and comprised articles and reports including published and unpublished dissertations, database, and it comprised articles and reports, including published and unpublished dissertations. All plague literature in Indonesia (1923–2019) was used as a reference for this article. The main data sources selected were reported from the Directorate General of Disease Prevention and Control, the Ministry of Health of Republic of Indonesia, Institute for Vector and Reservoir Control Research and Development (IVRCRD) Salatiga, the Ministry of Health of the Republic of Indonesia, Provincial Health Offices (West Java, Central Java, East Java, and Special province of Yogyakarta), PubMed, Google Scholar, Science Direct, Medicus Index for the Southeast Asian Region (IMSEAR). Bibliographic researches were conducted in Dutch, English, and Indonesian. Library search used Boolean operators [10].

## Satellite imaging

The Global Land Cover 1992–2020 database was used to monitor land cover changes. This is a database of global land cover data produced by a program of the European Space Agency. This database does not require any post-processing. It can therefore be used directly to visualize changes in land cover at a medium scale (spatial resolution of 300 m). A second database, Esri Sentinel-2 Land Cover Explorer, created by ESRI (Environmental Systems Research Institute, Redland, California, USA) using Sentinel-2 satellite imagery was used. This database shows global land cover change between 2017 and 2023, using only 2023 data. Seven land cover classes were identified: Water, Forest, Flooded fields, Fields, Urban, Bare ground, and Pasture.

## History of plague in Indonesia

**Fig 2:**
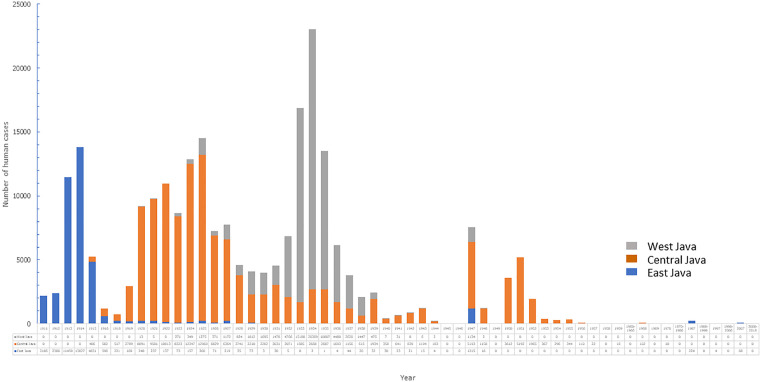
Human plague cases in Indonesia, 1911–2018.

In 1905, two confirmed cases of workers at the Port of Bandar Deli, East Coast of Sumatra (Sumatra Oostkust) were reported as the first plague cases in Indonesia [9]. However, the disease disappeared without any action [9]. Plague was again reported in Java in 1910. The bubonic plague in Java killed 245375 people between 1910 and 1960, with the largest number of deaths occurring in Central Java (51.5%), followed by West Java (17.6%), and East Java (3.9%) (Fig 1). Between 1920 and 1927, the number of deaths ranged from 800 to 10,000 per year [11]. The number of cases and deaths decreased from 1928 to 1931. The highest peak of cases and mortality occurred in 1934 with 23,267 cases and 23,239 deaths (Fig 2) [11]. Comprehensive plague control was then conducted by systematic reconstruction of rural dwellings and intensive vaccination. The program started in 1935. However, information and progress of the plague control program were not available during World War II. Plague surveillance resumed after the war. Plague was reported with a relatively high number of cases, i.e., 3,422 confirmed cases with 3,365 deaths in 1948, and 874 cases with 844 deaths in 1949. Cases were mostly found in Yogyakarta and Central Java, whereas in East and West Java, plague sufferers were not reported. No plague cases were reported until the second half of 1952 [6,9,12,13]. Plague cases were still found in several endemic areas in Java until the end of the 1960s. The disease then re-emerged between 1968 and 2007 as sporadic bubonic plague outbreaks in the Boyolali-Central Java and Pasuruan-East Java after a long period of silence [9].

## Characteristics of *Yersinia pestis* in Indonesia

*Y. pestis* is a gram-negative, non-motile, coccobacillus bacterium that belongs to the family Enterobacteriaceae. This bacterium has been isolated in Indonesia since 1968 and has been identified as *Y. pestis* var. *orientalis*. The identification of this strain is based on its ability to reduce nitrate to nitrite but not to ferment glycerol. The other characteristics of this strain of *Y. pestis* are the presence of fraction 1, VW antigen, and the presence of pesticin I-coagulase fibrinolytic complex. This strain also displayed pigmentation. *Y. pestis* var. *orientalis* spread worldwide during the third pandemic plague around 1855. The bacterial strains from Java displayed significant differences in protein patterns compared to *Y. pestis* strains from Asia and America based on gel electrophoresis. This strain carried two plasmids, pFra and pPla, which are important for the infection of mammalians. This bacterium can avoid the host’s immune response when the bacterium infects its host. The bacterium isolates of *Y. pestis* can be divided into four ribotypes (B, Q, R, and T). The most common is B, while the other three are located in Madagascar. In addition, molecular genetic analysis data, such as MLVA-25, are needed to better understand the dynamic of plague transmission in Indonesia and its links to global patterns. However, published MLVA-25 genotyping data for *Y. pestis* strains from Indonesia are extremely limited in international databases and the scientific literature. Only a few studies have included Indonesian isolates like Java-9 and Java-10, but these are sparse and do not provide a comprehensive picture of the strains currently circulating in Indonesia [14–19].

## Plague in East Java

The plague first occurred in East Java in October 1910, following the Dutch Indies colonial government’s decision to import rice from Rangoon (now Yangon), Burma (now Myanmar), in response to the rice crisis in Java. They also imported rats in the ship cargo. This first plague outbreak is a typical example of accidental introduction since an epidemic of bubonic plague was spreading in Burma at the same time. Rice was transported by ship to the Surabaya seaport, then distributed by train to Malang, East Java. However, the train circulation was cut off due to flooding between Wlingi, Blitar, and Malang. The rice was then stored in a nearby warehouse. Rats fed on the stored grains and as they were infested with contaminated fleas, many dead rats were found near the rice warehouse. Owing to the massive death of rats, fleas went to humans triggering the first plague outbreak in Indonesia. The first outbreaks of plague occurred in the villages of Dampit and Turen in Malang. Seventeen deaths were reported. From 1910 to 1912, the bubonic plague spread to Kediri, Surabaya, as well as Malang, with approximately 2,000 people infected [6,8,11,12]. The plague then spread to other districts in East Java such as Blitar, Tulungagung, and Madiun with a total of 177 confirmed cases [6,20,21]. The plague outbreak re-occurred East Java in 1986 after a quiescent period of 39 years. A total of 24 confirmed cases and 20 deaths were reported in Surolowo, Pasuruan district. In 1987, plague cases were recorded at the same place with 224 cases and one case of death. Six plague cases with no fatalities were confirmed in 1997 [9]. After 10 years of silence, bubonic plague cases were reported in Surolowo, East Java. A total of 68 cases (with one case of death) were reported in 2007. Since then, no cases of bubonic or pneumonic plague were reported in humans and no *Y. pestis* was found in rats and fleas [9].

## Plague in Central Java and Yogyakarta

Plague entered Central Java for the first time through the Semarang seaport in 1916 [12,22]. The epidemic then spread to Semarang and Ungaran with 76 confirmed cases. A subsequent epidemic was reported in some villages surrounding the Sindoro, Merbabu, and Merapi volcanoes from 1916 to 1918 [23,24]. This epidemic also affected populations in Surakarta District, Central Java (1,901 cases) and Yogyakarta in 1915 and 1916 [9,21]. Sporadic cases of plague were also reported at the Tegal seaport in 1927. Following 8 years of silence, the epidemic of plague resumed in Central Java and Yogyakarta from 1935 to 1938 [25]. The majority of deaths occurred in Central Java where 1,835, 1,246, 412, 473, and 2 deaths were reported in Tegal, Pekalongan, Wonosobo, Purbolinggo, and Surakarta, respectively. Meanwhile, Yogyakarta reported 118 deaths during this same period [25]. In 1968, plague cases were again recorded in Selo and Cepogo (Boyolali District in Central Java), two highly populated sub-districts situated on the slopes of the Merbabu and Merapi volcanoes at an altitude of about 1,600 m. A total of 102 human plague cases were reported, with 43 deaths occurring between 1968 and 1970. Most cases were found in highland areas which were difficult to access and had very limited medical facilities [6,14]. Other plague outbreaks occurred in Boyolali in 1970 with 11 cases and 3 deaths [26]. Since this last outbreak in 1970, no plague cases have been reported in Central Java [6,26].

## Plague in West Java

Plague was first reported in West Java when a ship from Indochina loaded with rice docked at the sea port of Cirebon in December 1921. It was a separate accidental introduction. A plague epidemic followed which affected Cirebon but also Kuningan, Majalengka, Ciamis, Tasikmalaya, Bandung, and Garut from 1921 to 1929 (Fig 2). Up to 1935, as many as 20,597 cases were recorded in West Java out of which 20,569 were fatal. Furthermore, sporadic outbreaks occurred in several districts of the West Java province from 1935 to 1938. Deaths were reported in Bandung (4,236), Garut (4,295), Sumedang (877), and Tasikmalaya (1,514) [25]. Since then, no cases have been reported in West Java.

## Plague vectors in Indonesia

The oriental rat flea, *X. cheopis*, was identified and confirmed as the principal vector of plague in Indonesia. This flea is known to transmit the bacteria from infected humans and/or animals to other warm-blooded humans and/or animals through their bites [27]. Another flea species capable of transmitting the bubonic plague, *Stivalius cognatus*, was identified in Java [6]. Both *X. cheopis* and *S. cognatus* were shown to spread plague among rats in Boyolali, one of the most important plague endemic areas in Central Java [9,25].

## Rodent-flea diversity associated with plague transmission in Indonesia

A total of 18 species of rats and 44 species of fleas have been identified in endemic bubonic plague areas in Java (Table 1). However, only 12 rat species and 4 flea species are known to play an important role in the transmission of bubonic plague [28–30]. Three rodent species, i.e., *Rattus tanezumi*, *Rattus exulans*, and *Rattus tiomanicus* were responsible for maintaining *Y. pestis* in nature. These rat species carry the bacterium in their blood or the peripheral layer of the body in sufficient quantities to transmit to other hosts. The peripheral layer of the body refers to the outer surface of the rat’s body, where the initial infection occurs. The peripheral layer of the body consists of the skin, subcutaneous tissue, and peripheral blood vessels (such as capillaries in the tail, ears, and feet) [25]. The house rat, *R. tanezumi* was found everywhere (villages, fields, and forests) during the plague outbreaks in Indonesia [25]. The population of this species is growing rapidly because it can breed indoors throughout the year with an average gestation period of 21 days and an average reproductive size of 5.4 (data recorded in Madagascar). In contrast, *Rattus norvegicus* breeds poorly in large cities. However, this species has been reported to have spread and reproduced in rural areas since the 1950s [25]. The susceptibility of rats to *Y. pestis* has been investigated in the Boyolali District in Central Java and the Pasuruan District in East Java [14,30,31]. *Rattus exulans* was quite easily infected with *Y. pestis*, but strongly resisted the disease. The house rat, *R. tanezumi,* was susceptible to the infection. The susceptibility and immunity/resistance of rats to *Y. pestis* seem to be influenced by the relationship between rats and their environment [5].

**Table 1:** Species of rats and fleas found in the plague enzootic area in Java, 1923–2019.

No.	Rats species fleas species	References									
Van Stenis (1923)	Otten (1924)	Schuurman (1930)	Swellengrebel al. (1950)	Baltazard and Bahmanyar (1960)	Turner et.al. (1974, 1975)	Hudson et.al. (1973)	Lim Boo Liat et.al. (1980)	Kusharyono et.al (1980)	Ristiyanto et.al. (2010–2018)
	**Rodentia**										
**1**	***R. tanezumi***	**X**	**X**	**X**	**X**	**X**	**X**	**X**	**X**	**X**	**X**
	*X. cheopis*	V	V	V	V	V	V	V	V	V	V
	*S. cognaus*	–	–	–	–	V	–	–	V	V	–
	*Neopsylla sondaica*	–	–	–	–	–	–	–	–	V	–
	*Pygiopsylla ahalae*	–	V	–	–	–	–	–	–	–	–
**3**	***Maxomys bartelsii***	**–**	**–**	**–**	**–**	**–**	**–**	**–**	**X**	**–**	**–**
**4**	***R. argentiventer***	**–**	**–**	**–**	**X**	**X**	**–**	**–**	**X**	**–**	**–**
	*X. cheopis*	–	–	–	–	–	–	–	V	–	–
	*Stivalius cognatus*	–	–	–	–	–	–	–	V	–	–
5	***R. norvegicus***	X	X	X	X	–	–	–	–	–	–
	*X. cheopis*	–	–	V	V	–	–	–	–	–	–
	*X. astia*	–	–	V	–	–	–	–	–	–	–
**6**	***Bandicota indica***	**–**	**X**	**–**	**–**	**X**	**–**	**–**	**–**	**–**	**–**
	*X. cheopis*	–	–	–	–	–	–	–	–	–	–
	*Pygiopsyla ahalae*	–	V	–	–	–	–	–	–	–	–
7	***Chiropoddyis gliroides***	X	X	–	–	X	X	–	–	–	–
	*X. cheopis*	–	–	–	–	–	–	–	–	–	–
	*Pygiopsylla ahalae*	–	V	–	–	–	–	–	–	–	–
8	***Mus muscullus***	–	X	–	–	–	–	–	–	–	–
	*X. cheopis*	–	–	–	–	–	–	–	–	–	–
**9**	***R. tiomanicus***	**–**	**–**	**–**	**–**	**–**	**X**	**–**	**X**	**X**	**X**
	*X. cheopis*	–	–	–	–	–	X	–	V	V	–
	*S. cognatus*	–	–	–	–	–	–	–	V	V	–
	*Neopsylla sondaica*	–	–	–	–	–	V	–	–	V	–
	*C. bartelsi*	–	–	–	–	–	–	–	–	–	–
**10**	***R. exulans/M. concolor***	**X**	**X**	**–**	**–**	**X**	**X**	**X**	**X**	**X**	**X**
	*X. cheopis*	–	V	–	–	–	V	–	V	V	V
	*X. astia*	–	–	–	–	–	–	–	–	–	–
	*S. cognatus*	–	–	–	–	V	V	V	V	V	V
	*Neopsylla sondaica*	–	–	–	–	–	V	–	–	V	X
	*Pygiopsylla ahalae*	–	V	–	–	–		–	–	–	–
**11**	***Niviventer bukit***	**–**	**–**	**–**	**–**	**–**	**X**	**–**	**X**	**X**	**X**
	*Stivaius cognathus*	–	–	–	–	–	V	–	–	V	V
**12**	***N. fulvesens/M. jerdoni***	**–**	**X**	**–**	**–**	**–**	**–**	**–**	**–**	**–**	**X**
	*S. jacobsoni*	–	–	–	–	–	–	–	–	–	V
	*N. sondaica*	–	–	–	–	–	–	–	–	–	V
	**Insectivora**	–	–	–	–	–	–	–	–	–	–
**13**	***Suncus murinus***	**–**	**–**	**–**	**–**	**X**	**–**	**–**	**X**	**X**	**–**
	*X. cheopis*	–	–	–	–	–	–	–	V	V	–
**14**	***Martes flavigata***	**–**	**–**	**–**	**–**	**–**	**–**	**–**	**X**	**–**	**–**
**15**	***Crocidura coerulea***	**X**	**X**	**–**	**–**	**–**	**–**	**–**	**–**	**–**	**–**
**16**	***C. renticola***	**–**	**–**	**–**	**–**	**–**	**–**	**–**	**X**	**–**	**–**
**17**	***Helomys suillus***	**–**	**–**	**–**	**–**	**–**	**–**	**–**	**X**	**–**	**X**

The resistance of house rats to *Y. pestis* infection can be transmitted to the offspring [31]. This trait was also found in *Mastomys natalensis* mice in Southern Africa [32]. House rats are resistant to *Y. pestis* due to deletion of 32 base pairs in the chemokine receptor 5 (CCR5) gene. Laboratory tests on healthy rats have shown that the CCR5-Δ32 receptor does not guarantee to be infected by *Y. pestis* [31,33]. However, the unique substitution (H184R) in the CCR5 gene was found to be more common in resistant rats compared to susceptible ones. This trait is also more common in rats from plague-foci areas than from the plague-free regions [34]. The presence of both sensitive and resistant rats allows *Y. pestis* to be naturally maintained [35,36]. However, the immune response to infection might be different even for the same rat species in the same endemic area. The presence of rats resistant to *Y. pestis* may explain the occurrence of periods of silence [37].

## Environmental and climate changes in Indonesia

**Fig 3:**
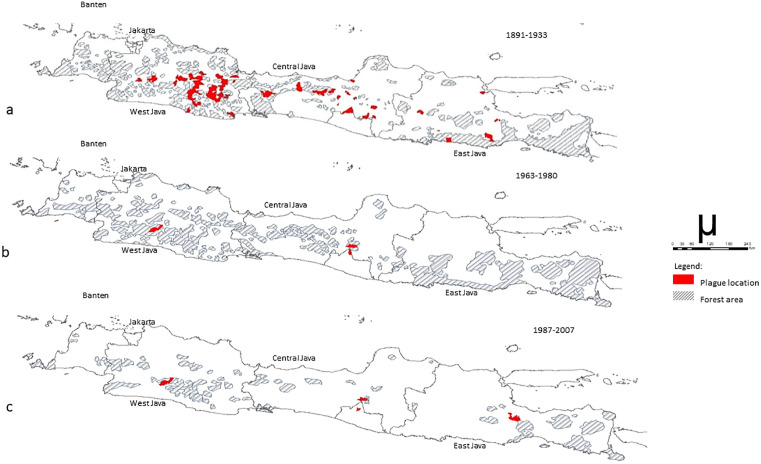
Forest areas in Java at the time of plague outbreaks. **a.** Forest area in 1910 and Plague cases distribution around 1891–1933 in Java. **b.** Forest area in 1963 and Plague cases distribution around 1963–1980 in Java. **c.** Forest area in 1987 and Plague cases distribution around 1987–2007 in Java. Map base layer: https://maps.arcgis.com/home/item.html?id=4ad930360ef64a828cf58a1a127c9a9d.

Bubonic plague has been reported both in cities and villages, and even in lowlands, such as Surabaya, Malang, Semarang, and Cirebon from 1911 to 1947. Since 1968, *Y. pestis* has been isolated from fleas in rural areas and hilly areas more than 500 m above sea level. Although bubonic plague is not reported, *Y. pestis* is thought to still circulate among wild rats and rodents in endemic areas or plague enzootic regions. The plague enzootic area in Java covers the Pasuruan District-East Java, located in the Tutur Village, Nongkojajar sub-district, which is a mountainous area at an altitude of 1,200–1,400 m, and the Bromo-Tengger National Park. The plague enzootic areas in Selo and Cepogo Subdistrict, Boyolali District in Central Java; and Cangkringan Subdistrict and Sleman District in Yogyakarta are located on the slopes of Mount Merapi with elevations between 200 and 1,500 m which includes the Merapi-Merbabu forest area. In West Java, the plague enzootic area is located in the Ciwidey Subdistrict, Bandung District, and the Ciremai mountains. Changes in the distribution of plague in Indonesia were expected due to intensive surveillance of humans, rats, and fleas along with sanitation, education, economy, and population awareness [18,27]. Furthermore, the land use within plague enzootic areas has changed significantly over the past 100 years. Between 1898 and 1937 the island of Java lost 22,000 km^2^ of natural forest. Around 1939–1945, 23% of Java was still covered by natural forests, in 1973 it shrank to 11%, and by 1990, the area of Java covered by forests was only 7%, or 0.96 million hectares. According to Pavlovsky [38,39], in Siberia, the conversion of forests into agricultural areas had a significant effect on the decline in cases of plague. Global warming is another factor to consider. The average temperature of the Earth surface has risen by 0.74 ± 0.18 °C over the last 100 years and is expected to continue rising. Changes in global climate can affect the distribution pattern and cycle time of transmission of plague [40] due to changes in the distribution of rats and fleas. This could either potentially limit its development or trigger emergence in new areas. Thus, between 1870 and 2020, the average temperature in Indonesia increased by 1.2°, rising from 25.8° to 27° (Fig 3). This rise in temperature may be conducive to the development of fleas. Furthermore, large-scale climate phenomena such as the El Niño Southern Oscillation (ENSO) and the Indian Ocean Dipole (IOD) interact with each other to influence disease via precipitations and temperature.

## Environmental changes in Java

**Fig 4:**
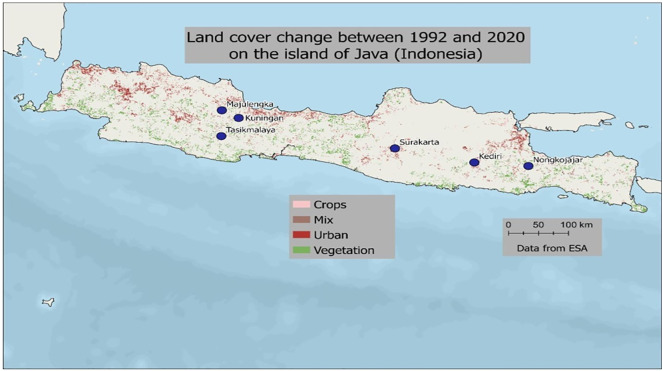
Land cover change between 1992 and 2020 on the island of Java. Data from ESA. Land Cover CCI Product User Guide Version 2. Tech. Rep. (2017). Available at: https://maps.elie.ucl.ac.be/CCI/viewer/download/ESACCI-LC-Ph2-PUGv2_2.0.pdf. Map base layer: https://maps.arcgis.com/home/item.html?id=4ad930360ef64a828cf58a1a127c9a9d.

Between the 1950s and 1980s, Java underwent a profound environmental transformation due to deforestation. The surface of forested areas varied between each series of plague outbreaks (Fig 3a–3c). Fig 4 shows the changes in urbanization and vegetation cover on Java Island between 1992 and 2020. Table 2 shows the percentage of change in the main land use categories (cropland, vegetation, and urban areas) between 1992 and 2020 for the six locations where the plague occurred in the last century. Throughout these six locations, with the exception of Nongkojajar, which has remained very rural, agriculture, and vegetation have declined in favor of urbanization which may increase the risk of contact between fleas and humans.

**Table 2:** Changes in the main land-use categories between 1992 and 2020 for locations where the plague occurred in the last century.

	LLUC 1992–2020	
	Kediri	Tasikmalaya	Surakarta	Nongkojajar	Majalengka	Kuningan
Cropland (number of pixels)	−85	−42	−128	−6	−61	−34
Cropland/Vegetation (number of pixels)	−26	−68	−17	−10	−1	−19
Vegetation (number of pixels)	−4	−122	−71	14	−4	−41
Urban (number of pixels)	116	232	217	2	66	94
Cropland (km2)	−7.65	−3.78	−11.52	−0.54	−5.49	−3.06
Cropland/Vegetation (km^2^)	−2.34	−6.12	−1.53	−0.9	−0.09	−1.71
Vegetation (km^2^)	−0.36	−10.98	−6.39	1.26	−0.36	−3.69
Urban (km^2^)	10.44	20.88	19.53	0.18	5.94	8.46
Cropland (%)	−20.00%	−30.66%	−58.99%	−7.79%	−13.17%	−17.71%
Cropland/Vegetation (%)	−17.81%	−18.66%	−85.00%	−2.80%	−0.51%	−5.37%
Vegetation (%)	−28.57%	−61.33%	−63.39%	3.45%	−7.55%	−4.44%
Urban (%)	46.40%	166.91%	44.93%	No urban	60.55%	1342.86%

**Fig 5:**
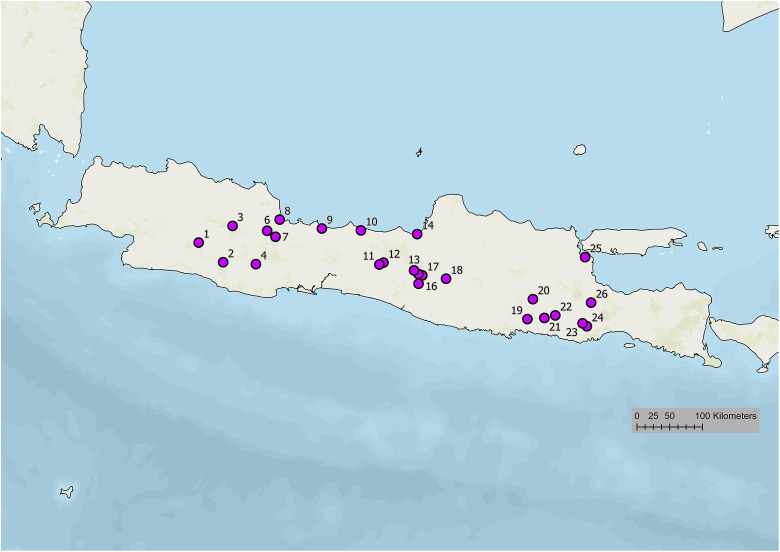
Spatial distribution of the 26 plague outbreaks on the island of Java between 1905 and 2007. 1 Rawabogo-Ciwidey, Bandung, West Java; 2 Garut, West Java; 3 Sumedang, West Java; 4 Tasikmalaya, West Java; 5 Ciamis, West Java; 6 Majalengka, West Java; 7 Kuningan, West Java; 8 Cirebon, West Java; 9 Tegal, Central Java; 10 Pekalongan, Central Java; 11 Wonosobo, Central Java; 12 Mount Sindoro, Central Java; 13 Mount Merbabu, Central Java; 14 Semarang, Central Java; 15 Selo-Boyolali, Central Java; 16 Cangkringan-Sleman, Yogyakarta; 17 Cepogo-Boyolali, Central Java; 18 Surakarta, Central Java; 19 Tulungagung, East Java; 20 Kediri, East Java; 21 Blitar, East Java; 22 Wlingi-Blitar, East Java; 23 Turen-Malang, East Java; 24 Dampit-Malang, East Java; 25 Surabaya, East Java; 26 Tutur-Nongkojajar, Pasuruan, East Java. Map base layer: https://maps.arcgis.com/home/item.html?id=4ad930360ef64a828cf58a1a127c9a9d.

**Fig 6:**
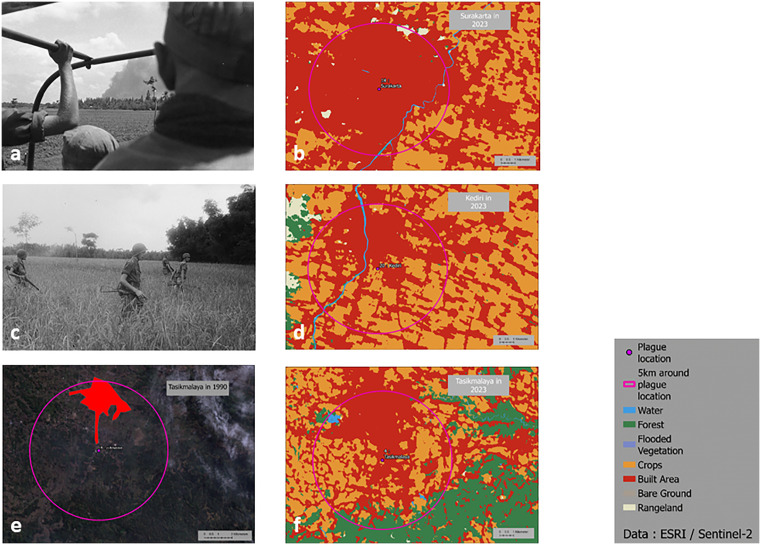
Landscape evolution in sites of plague outbreaks. **a.** Surakarta in 1948 (site 18) (Source: Nationaal Archief). **b.** Land use in Surakarta in 2023 (Source: ESRI/Sentinel-2). **c.** Urban area of the Tasikmalaya focus in 1990 (Source: Landsat 5). **d.** Land use in Tasikmalaya in 2023 (Source: ESRI/Sentinel-2). **e.** Cornfield in Kediri around 1948 (Source: Nationaal Archief). **f.** Land use in Kediri in 2023 (Source: ESRI/Sentinel-2). Map base layer: https://maps.arcgis.com/home/item.html?id=4ad930360ef64a828cf58a1a127c9a9d.

**Fig 7:**
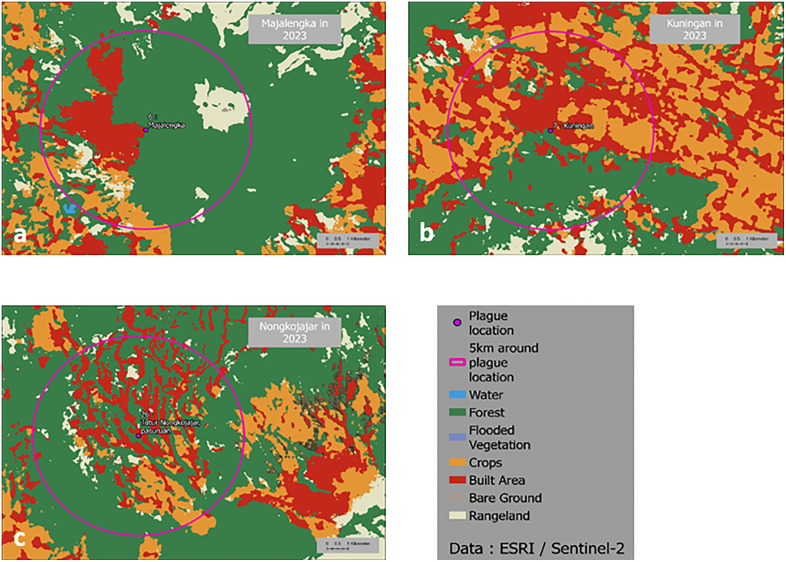
Current landscape in former sites of plague outbreaks. **a.** Land use in Majalengka in 2023 (Source: ESRI/Sentinel-2). **b.** Land use in Kuningan in 2023 (Source: ESRI/Sentinel-2). **c.** Land use in Nongkojajar in 2023 (Source: ESRI/Sentinel-2). Map base layer: https://maps.arcgis.com/home/item.html?id=4ad930360ef64a828cf58a1a127c9a9d.

Four kinds of environmental modification could be identified within 26 historical locations of plague in the island of Java (Fig 5 and Table 3). The first type of land-use transformation corresponds to half of the locations, i.e., 13. These sites are distributed in West Java (sites 3, 4, 5, and 8), Central Java (sites 9, 10, 14, and 18), and East Java (sites 19, 21, 23, 24, and 25). These are places where the urban population is growing and the rural population is shrinking, and where living and housing conditions have generally improved. In these locations, regular contacts with rats might be considered less likely at first glance. However, some rat species, in particular house rats, accommodate very well and thrive in cities, especially when waste management is not well conducted. Better living conditions combined with municipal waste disposal tend to reduce the risk. This concerns, for instance, Tasikmalaya in West (site 4), where the urban area has expanded over the agricultural land between 1990 (Fig 6a) and 2023 (Fig 6b). As a result, the southern part of the city, which was previously covered by fields is now used for shops and housing. Another example is Surakarta in Central Java (site 18) which was an agricultural land in 1948 (Fig 6c) and is now largely urbanized (Fig 6d). The second type of environmental alteration corresponds to a combined growth in both urban and rural environments at the expense of the forest. This reorganization is mostly observed in Central Java (sites 11, 12, 13, 15, 16, and 17). Only one site is found in Eastern Java, Kediri (site 20), while none are found in Western Java. Kediri was mostly devoted in 1948 to the growth of maize (Fig 6e). Although, it is still the case today, fields have been pushed away by urban development. This creates interfaces between housing and fragmented agricultural areas (Fig 6f). The third configuration concerns areas where there has been little change in land use over time. These sites, i.e., 1, 2, 6, and 7, are located in Western Java (Fig 7a and 7b). If there was a risk of plague outbreak in the past, it might still be present since nothing has changed. These sites correspond to urban settlements directly bordering the forest, such as in Majalengka (Fig 7a) or Kuningan (Fig 7b). The last category of landscape transition corresponds to only one site in Eastern Java, i.e., site 26, in Tutur-Nongkojajar (Fig 7c). This site corresponds to the latest outbreak of plague in Indonesia in 2007. This site is very close to the forest at the boundary between woods and cleared areas (Fig 7c).

**Table 3:** Plague outbreaks in Indonesia.

Province	District	Location	Coordinates	Year of plague outbreaks	Note
West Java	Bandung	Rawabogo	−7.066410, 107.434495	1921–1929; 1935–1938	Bubonic plague cases were confirmed
West Java	Garut	Garut	−7.335254, 107.769883	1921–1929; 1935–1938	Bubonic plague cases were confirmed
West Java	Sumedang	Sumedang	−6.838426, 107.899045	1935–1938	Bubonic plague cases were confirmed
West Java	Tasikmalaya	Tasikmalaya	−7.362017, 108.218843	1921–1929; 1935–1938	Bubonic plague cases were confirmed
West Java	Ciamis	Ciamis	−7.326521, 108.319965	1921–1929	Bubonic plague cases were confirmed
West Java	Majalengka	Majalengka	−6.904645, 108.373193	1921–1929	Bubonic plague cases were confirmed
West Java	Kuningan	Kuningan	−6.986589, 108.488290	1921–1929	Bubonic plague cases were confirmed
West Java	Cirebon	Cirebon	−6.751038, 108.544764	1921	First bubonic plague outbreak in West Java
Central Java	Tegal	Tegal	−6.873466, 109.123075	1935–1938	Bubonic plague cases were confirmed
Central Java	Pekalongan	Pekalongan	−6.899679, 109.658108	1935–1938	Bubonic plague cases were confirmed
Central Java	Wonosobo	Wonosobo	−7.367761, 109.910802	1935–1938	Bubonic plague cases were confirmed
Central Java		Villages around Mount Sindoro	−7.341152, 109.967630	1916–1918	Bubonic plague cases were confirmed
Central Java		Villages around Mount Merbabu	−7.448069, 110.386613	1916–1918	Bubonic plague cases were confirmed
Central Java	Semarang	Semarang	−6.950367, 110.429311	1916	First bubonic plague outbreak in Central Java
Yogyakarta	Sleman	Cangkringan	−7.629982, 110.448351	1915, 1916	Bubonic plague cases were confirmed
Central Java	Boyolali	Cepogo	−7.516080, 110.499303	1916–1918, 1968	Bubonic plague cases were confirmed
Central Java	Boyolali	Selo	−7.495874, 110.445257	1916–1918, 1968	Bubonic plague cases were confirmed
Central Java	Surakarta	Surakarta	−7.561930, 110.825506	1935–1938	Bubonic plague cases were confirmed
East Java	Tulungagung	Tulungagung	−8.114652, 111.940952	1910–1912	Bubonic plague cases were confirmed
East Java	Kediri	Kediri	−7.842074, 112.015839	1910–1912	Bubonic plague cases were confirmed
East Java	Blitar	Blitar	−8.099072, 112.172121	1910–1912	Bubonic plague cases were confirmed
East Java	Blitar	Wlingi	−8.065158, 112.322862	1910	Many dead rats were found for the first time
East Java	Malang	Turen	−8.173063, 112.696660	1910	First bubonic plague outbreak in Indonesia
East Java	Malang	Dampit	−8.213237, 112.755893	1910	First bubonic plague outbreak in Indonesia
East Java	Surabaya	Surabaya	−7.266076, 112.731218	1910–1912	Bubonic plague cases were confirmed
East Java	Pasuruan	Tutur-Nongkojajar	−7.889525, 112.812743	1986, 1987, 1997, 2007	Bubonic plague cases were confirmed

## Ecology and biology of rats and fleas in the plague enzootic area

The risk of plague transmission to humans is dependent on the biology, ecology, interactions, and survival dynamics of rodents and fleas. Even though human cases of plague have not been found for more than a decade, four districts in Java are still marked as plague focus areas in Indonesia, i.e., Pasuruan District in East Java, Boyolali District in Central Java, Sleman district in Yogyakarta and Bandung district in West Java [1,13,25,29]. In Pasuruan District, plague cases in humans were reported in 1986, 1987, 1997, and 2007, with a case fatality rate reaching 83.7%. *Y. pestis* has also been detected in fleas and rats. House rats (*R. tanezumi*), Polynesian rats (*R. exulans*), tree rats (*R. tiomanicus*), chestnut white-bellied rats (*Niviventer fulvescens*), red spiny rats (*Maxomys surifer*), rice field rats (*R. argentiventer*), Javan short-tailed gymnures (*Hylomys suillus*), and Asian house shrews (*Suncus murinus*) are among the species of rodents and shrews that are frequently observed in this area. The general flea index averaged 1.7 [13,25]. Interestingly, the plague enzootic area in East Java displayed no significant changes in land use since the first plague outbreak was reported. This location remains a forest area with community plantations and settlements around the national park area. Changes occurred only in the structure of houses. More than 40 years ago, the buildings were predominantly made of woven bamboo and wood. Currently, most buildings are made of bricks or concrete blocks. Under these conditions, it is possible that the plague cycle in rodents continues with a potential to be transmitted to humans. Previous studies in East Java have also revealed that fleas carrying *Y. pestis* can live longer in rat burrows and infected rats when the right environmental factors are present, such as an optimum temperature of 28–30 °C. Several species of rat exhibit differing reactions to *Y. pestis* infection. Certain species, like *R. tanezumi*, may be more vulnerable to plague than other species. Populations of the same species of rat in different geographic locations may differ in their immunity to plague because some populations may result from genetic selection caused by past exposure to plague. *Rattus exulans*, *R. tiomanicus*, and *N. fulvescens* in Mount Bromo Tengger National Park, Pasuruan District in East Java, where plague outbreaks often occurred, might be more resistant to plague than the same rat species in Bandung District in West Java, Boyolali District in Central Java, or in Yogyakarta. There is a silent period during which the plague has the potential to spread to humans [13,25,29,41,42].

The plague enzootic area in Sleman District, Yogyakarta, is a hilly area located in forests and villages (Kepuharjo, Argomulyo, Glagaharjo, Umbul Harjo, and Wukirsari). An outbreak of bubonic plague was first reported in 1948. Positive serology of plague among rats was recorded in 1990, 1991, 1992, 1993, and 1996. However, *Y. pestis* was not detected in humans, rats, and fleas since 1997. This area has experienced significant land use changes, from forests and villages to more developed villages, tourist destinations, and agricultural areas. Plague surveillance activities for humans, rats, and fleas are still being carried out in plague enzootic areas in Yogyakarta. The results of field rat collections showed that the species of rats found were house rats (*R. tanezumi*), Polynesian rats (*R. exulans*), greater bandicoot rats (*Bandicota indica*), and house shrews (*Suncus murinus*). The house rat is the dominant rodent in the area. Meanwhile, the fleas often found in mice are *X. cheopis* and *Stivalius cognatus*, with an average general index of 1.02 [43].

The plague enzootic area in Boyolali District in Central Java is located in Selo and Cepogo villages. The last case of plague in humans was discovered in 1970, and since 1971, no cases of plague in humans have been reported while *Y. pestis* was not detected in rats and fleas. The monitoring of *Y. pestis* was done on house rats (*R. tanezumi*), Polynesian rats (*R. exulans*), chestnut white-bellied rats (*N. fulvescens*), red spiny rats (*Maxomys surifer*), and greater bandicoot rats (*B. indica*). The fleas often found in rats are *X. cheopis*, *S. cognatus*, and *Neopsylla sondaica*. The general flea index averaged 1.23. The environment in this area evolved from forests, rice fields, and villages, to more developed villages, some still being in forests, rice fields, plantations, and tourist destinations [44].

In West Java, the plague enzootic area is located in Bandung District, an area which a century ago was characterized by the presence of forests, villages, and agricultural land in the crater area of an extinct volcano (Kawah Putih). The last case of plague in humans in Bandung District was reported in 1930. Surveillance of plague in rats and fleas was carried out every year. The rat species most frequently found were house rats (*R. tanezumi*), tree rats (*R. tiomanicus*), brown rats (*R. norvegicus*), chestnut white-bellied rats (*Niviventer sp*.), red spiny rats (*M. surifer*), and house mouse (*Mus musculus*), while the mouse flea that is often found is *X. cheopis*, *X. brasiliensis*, and *S. cognatus* with an average general index of 0.43. This area has developed into a touristic, agricultural, and suburban area with a small remaining forest surface in the Kawah Putih volcano [45].

## Plague control in Indonesia

Since its introduction in Indonesia, the surveillance of bubonic plague cases and the monitoring of the presence of *Y. pestis* in rodents and fleas have been top priorities for the National Plague Control Program from 1923 to 2019. Updated information is critical for effective plague prevention and control programs, including the incidence and distribution of cases. The main objective of the plague control program is to reduce or maintain the plague morbidity and mortality rate at their lowest. Epidemiological measures include active and passive detection, isolation, quarantine, and treatment of the patients, laboratory evaluations (staining, culture, and pooling test), and vaccination. All confirmed or suspected plague cases were treated with antibiotics as prescribed by WHO [6,13,23]. Isolation and quarantine for plague cases were strictly implemented. No residents of a foci area (without exception) were allowed to leave until isolation was ended. Diseased people and all contact persons were isolated at the patient’s own home and were not allowed to leave the house. These measures have been implemented in Selo and Cepogo Subdistricts and Boyolali District in Central Java during the bubonic plague epidemic of 1968. In addition, the Boyolali District Government recommended that people raise their beds to more than 25 cm to prevent access from jumping fleas. The government also prohibited raising rabbits and guinea pigs in plague areas [46]. Between 1987 and 1995, in foci areas such as Sulorowo Village, Nongkojajar Subdistrict, and Pasuruan Subdistrict in East Java, tiled glass and ceramic floors have been installed in houses in order to facilitate cleaning.

According to WHO [47], the most efficient way of preventing plague outbreaks is the control of fleas rather than rodents. The most effective flea control method was dusting (sprinkling insecticide powder around the houses). The dusting method was applied during the outbreaks in 1968–1970, 1987, and 2007, in Boyolali District in Central Java and Pasuruan District in East Java, especially when the bubonic plague incidents occurred. The dusting method using insecticide-treated bamboo and PVC pipes. These methods could reduce the usage of insecticides by up to 90% and were effective in reducing the general index of fleas by more than 80% for 3 months. In addition, a fogging trial has been conducted in the Bulurejo Subvillage, Selo Subdistrict, and Boyolali District, covering all indoors and garden habitats using the insecticide malathion at 5%. However, this method was found less effective with no decrease in the general flea index [48]. Spraying was carried out in every case house and its surroundings in all endemic villages. In 1968, the Pasuruan Health Office sprayed 80 house units using Dichlorodiphenyltrichloroethane. However, these methods generate pollution and are susceptible to the buildup of insect resistance to pesticides [49].

## Eradication or silent period?

Laboratory confirmation is an essential step in the plague surveillance process. However, in Indonesia, only a few laboratories can analyze plague samples. The referral laboratory is located in Yogyakarta and the staff has been trained by US-CDC in 1995 to assess plague infection using both serology and bacteriology. The laboratory has the capacity for serological testing of blood samples (passive hemagglutination and passive hemagglutination inhibition methods) as well as bacteriological tests. However, due to decentralization, this laboratory is now under the jurisdiction of the Provincial Government of Yogyakarta with a limited budget. The laboratory is equipped with Biosafety Level (BSL) 1&2 laboratories, but the minimum authorized BSL level to maintain *Y. pestis* cultures is BSL-3. A major constraint is the lack of positive control sera, which were only received from US-CDC, so now no positive controls are used in plate evaluation. Another constraint is the limited production of F1 antigen due to a limited stock of *Y. pestis*. This referral laboratory for plague examination in BLK (Health Laboratory Center) in Yogyakarta needs to be improved to meet international standards, especially in infrastructure and facilities [49]. The Ministry of Health recently built another plague laboratory in Nongkojajar, Pasuruan, in East Java. These major technical constraints to the monitoring of plague and *Y. pestis* raise the question of whether there is a silent period of plague or a lack of detection of an ongoing circulation in Indonesia [49].

A major issue to consider is whether the environmental changes observed all over Java Island could lead to the eradication of plague by affecting rodent habitat. While one historical plague location remained mostly unchanged in terms of environment in East Java (Sulorowo Village, Nongkojajar Subdistrict, Pasuruan District), the other locations have undergone major environmental changes mostly leading to a higher presence of human populations and a higher human population density. The main vectors and rodent species involved in the historical episodes of plague are still present in all sites. Therefore, the potential risk is still here and the context might have been worsened. Indeed, with a higher human presence in these areas, the risk of contact human/rodents/fleas, and thus transmission to humans, is higher. Rodents are attracted to human houses and even if the constructions have improved in quality, rats are still present. One can just think of major cities or megacities worldwide. Although, built with stones and concrete, they are invaded by rats. One reason is that the development of cities goes along with the development of sanitation and sewage systems, which are very suitable environments for rats. A higher concentration of humans also means a more abundant source of food for the rats. Furthermore, human houses provide a safe environment for females to give birth far from their predators. All of these lead to an increased risk of contact and transmission. The absence of *Y. pestis* is questionable owing to the operational problems encountered by the referral laboratories. Therefore, a reasonable and conservative approach would be to consider that currently Indonesia is only experiencing a silent period of plague. Surveillance and monitoring should thus be a mandatory effort.

## Surveillance and monitoring

The Indonesian national plague response has been assessed by WHO and US-CDC in 2007 and 2019 at the initiative of the Ministry of Health of the Republic of Indonesia. The results of the evaluation by plague epidemiologists from CDC Fort Collins, United States, in 2007 showed that surveillance in humans, rats, and fleas had been carried out intensively in Boyolali District in Central Java Province and in Pasuruan District in East Java Province in order to anticipate any outbreak events. WHO and US-CDC decided that the classification of Indonesia as plague foci area, threatened and ex-plague area were irrelevant since no plague evidence could be found.

However, the referral laboratory for plague examination in BLK (Health Laboratory Center) in Yogyakarta needs to be improved to meet international standards [49]. Rodent and flea control should be carried out at least once a year along with active human surveillance and community education on plague control. Biosecurity and biosafety in laboratories must be upgraded while quality control system and laboratory quality assurance (both internal and external) need to be set up. Specific measures must also be taken such as improving specimen collection and transport (collection kit), standardization of laboratory, and optimization of techniques and procedures: ELISA (rodent and human), PCR (human, rodent, fleas), and rapid diagnostic test. Furthermore, the dissemination of information on plague control has been carried out by attaching posters at health centers, sub-district offices, and frequently visited public places in order for health workers and public to remain aware. Posters display plague control information, signs and symptoms of bubonic and pneumonic plague, modes of transmission, prevention, actions to take when symptoms are observed and the effects of plague. Those are some of the efforts needed to avoid any outbreaks during the silent periods of plague in Indonesia [49].

## Conclusion

The results and conclusions presented in this article are of a review and evaluative nature, unfortunately rigorous statistical validation is not feasible because the last plague epidemic took place in eastern Java in 2007. In fact, we are moving from 1968, when satellite images did not exist, to 2007, when the plague reappeared in the Tutur-Nogkjajar region, a place conducive to the emergence of the plague. That is why we have just selected land use data from 2023 and compared it to the few photographs available from the period when the cases occurred. Nevertheless, since the occurrence of the first outbreak of plague in Indonesia in 1910, the environment has changed in many parts of Indonesia due to the human population growth, land conversion, and urban extension. With the exception of Eastern Indonesia where the environment remained the same, land conversion strongly affected the landscape in other places of initial plague outbreaks. However, this land conversion did not affect the populations of rodents and fleas since the same species are still found. There is thus a risk of other outbreaks. The environmental changes did not result in an eradication of the risk of plague outbreaks. Furthermore, since the human population has increased and the populated areas have extended since the initial outbreaks, the risk of contact is statistically higher. The conclusion is therefore that the current phase of absence of plague outbreaks is more likely to be a silent period with a risk of re-emergence rather than an eradication.
